# Virus detection and identification using random multiplex (RT)-PCR with 3'-locked random primers

**DOI:** 10.1186/1743-422X-4-65

**Published:** 2007-06-28

**Authors:** Amy L Clem, Jonathan Sims, Sucheta Telang, John W Eaton, Jason Chesney

**Affiliations:** 1 Molecular Targets Program, Medical Oncology, J.G. Brown Cancer Center, University of Louisville, Kentucky, USA

## Abstract

**Background:**

PCR-based detection and identification of viruses assumes a known, relatively stable genome. Unfortunately, high mutation rates may lead to extensive changes in viral nucleic acid sequences making dedicated PCR primer use problematic. Furthermore, in bioterrorism, viral consensus sequences can be genetically modified as a countermeasure to RT-PCR and DNA chip detection. Accordingly, there is a great need for the development of rapid and universal virus detection and identification technologies.

**Results:**

We report herein that viral genomic DNA or RNA can be separated from host nucleic acids in plasma by filtration and nuclease digestion, and randomly amplified in a single PCR using a mixture of primers designed to be resistant to primer-dimer amplification (5'-VVVVVVVVAA-3', V = A, G or C; 3^8 ^or 6561 primers). We have termed this novel PCR method Random Multiplex (RT)-PCR since hundreds of overlapping PCR amplifications occur simultaneously. Using this method, we have successfullydetected and partially sequenced 3 separate viruses in human plasma without using virus-specific reagents (*i.e., *Adenovirus Type 17, Coxsackievirus A7, and Respiratory Syncytial Virus B). The method is sensitive to ~1000 genome equivalents/ml and may represent the fastest means of detection of unknown viruses.

**Conclusion:**

These studies suggest that the further development of random multiplex (RT)-PCR may lead to a diagnostic assay that can universally detect viruses in donated blood products as well as in patients suffering with idiopathic disease states of possible viral etiology.

## Background

Relatively benign viruses can be converted into highly virulent viruses via the introduction of genes of interest. For example, Ectromelia virus, a natural pathogen of mice that causes mousepox, recently was recombined with interleukin-4 as part of an effort to develop a live virus immuno-contraceptive vaccine. Surprisingly, the recombined virus caused 60% mortality in 2 strains of mice, whereas the wild type virus caused no death [1]. A credible bioterrorism scenario might entail the release of such a recombined or chimeric virus tailored for maximum infectivity and pathogenicity but not readily detectable using our current "state-of-the-art" diagnostics (*i.e., *PCR and micro-array chips.) Accordingly, there is a need for methods that can identify unknown viral pathogens and which can reveal extensive genomic information. Such methods would not only prove useful for our defense against bioterrorism, but also would improve our capacities to identify and control outbreaks of naturally occurring pathogenic viruses.

The proven technology to rapidly detect and identify known human pathogens as potential causes of disease or as bioweapons is PCR [2]. A recent example involved a case of fatal yellow fever (YF) in a traveler returning from Amazonas, Brazil in March, 2002 [3]. The otherwise healthy 47-year-old male developed fever, headache, pancytopenia, coagulopathy, renal and liver failure. Pan-cultures were negative, and a peripheral smear yielded no plasmodia. Serological tests (IgG, IgM) performed at the CDC were negative for YF, dengue, St. Louis encephalitis, and other agents, but serum specimens (unfortunately examined only post-mortem) were positive for YF viral RNA as measured by RT-PCR [3]. Subsequent attempts to isolate or culture the virus failed. This example highlights the superiority of PCR over other currently available methods. DNA chips that allow the simultaneous measurement of literally thousands of genes through hybridization are now being developed as the next-generation rapid diagnostic test for all known human pathogens [4]. However, both of these technologies rely on a relatively stable genome, and several human pathogens display a high mutation rate (*e.g., *HIV 2 × 10^-5^/base, 9 kB genome [5]). Moreover, the ability to recombine "non-pathogenic" viruses *in vitro *introduces the potential not only for *de novo *pathogenicity but also for enhanced stealth. These considerations suggest that it may be impossible to design DNA micro-arrays which detect nucleic acids from all known and unknown viruses, including less obvious vehicles of bioterrorism such as adenovirus or rhinovirus recombined with a single gene for enhanced virulence. Accordingly, there is a great need for the development of techniques that enable the universal amplification of viral nucleic acids.

There are 2 strategies to amplify genetic sequences with PCR without prior knowledge of the precise sequences. The first strategy relies on the degenerate binding of arbitrarily chosen primers to sample multiple cDNAs or genomic DNA species during PCR (two related methods are known as differential display and RNA Arbitrarily Primed PCR [RAP-PCR] [6-8]). The amplicons range in size from ~50–600 bp and overlap. RAP-PCR was recently used successfully to identify a novel human pneumovirus only after the virus had been cultured [9]. These techniques yield an amplicon "fingerprint" and are generally used to compare two populations of nucleic acids. Accordingly, the pneumovirus was identified by comparing infected cells with non-infected cells [9]. The other strategy is known as Sequence-Independent, Single-Primer Amplification (SISPA) and involves the directional ligation of a linker/adaptor oligonucleotide onto both ends of a target population of either DS DNA or DS cDNA (after reverse transcription from RNA) [10]. The PCR is accomplished with primers specific for the linked adaptor molecule. SISPA, followed by extensive immunoscreening, enabled the cloning of the Norwalk agent genome from stool and Hepatitis G virus from plasma [11]. Unfortunately, these techniques have not found great utility in the direct discovery or identification of novel viruses from human plasma or serum samples (unless accompanied by immunoscreening) because of the relatively high level of host genomic DNA. However, recent studies indicate that DNAse treatment of serum samples can degrade most of the host genomic DNA but not affect viral nucleic acids, which are protected by stable capsids [12]. The combination of DNAse treatment with SISPA was successfully used to identify and sequence in their entirety 2 novel parvoviruses from bovine serum [12].

While the aforementioned PCR techniques are useful for the discovery of novel viruses, their utility to rapidly detect and identify unknown (or recombined) viruses is quite limited. RAP-PCR, random RT-PCR and SISPA are complicated multi-step procedures requiring infection of cultured cells, days to accomplish, expensive reagents and broad technical expertise. Accordingly, these methods have not found clinical utility for the rapid detection or identification of viral agents.

Here, we present a novel method for rapid virus detection and identification based on random multiplex (RT)-PCR using 3'-locked random primers to avoid primer-dimer amplification. Once detected, virus amplification products can be shot-gun cloned and sequenced for identification. This method may prove useful for the rapid detection and identification of emerging or recombined viruses.

## Results

### Filtration and Nuclease Treatment Improves Viral DNA Amplification by Removing Host DNA and mRNA

**Figure 1:**
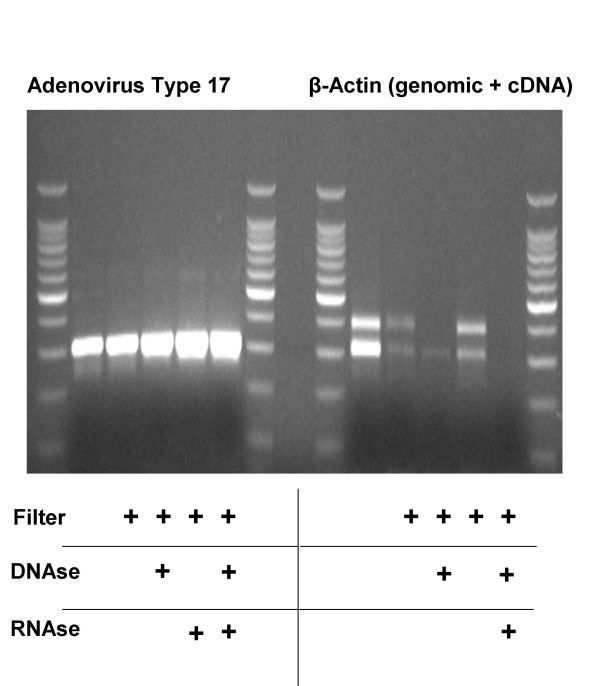
**Filtration and Nuclease Treatment Improves Viral DNA Amplification by Removing Host DNA and mRNA**. Human plasma was inoculated with 1 μl of an Adenovirus Type 17 suspension, filtered and incubated for 2 hrs with either 10 u DNAse or 5 u RNAse as indicated. Remaining nucleic acids were purified with the QiaAmp UltrasSens Virus Kit and subjected to 1^st ^strand cDNA synthesis and 50 cycles PCR using primers specific for either Adenovirus Type 17 or human β-Actin DNA (upper band; primers cross an intron) or cDNA (lower band). Amplicons were then visualized on an ethidium bromide impregnated agarose gel.

We hypothesized that random amplification of viral nucleic acids isolated from human plasma might provide a powerful new method to detect and identify novel and recombined viruses. Allander *et al. *recently found that subjecting bovine plasma to filtration (to remove cells) and DNAse treatment (to remove genomic DNA) was sufficient to separate viral nucleic acids from bovine nucleic acids and thus enable SISPA and cloning of novel viruses [12]. The mechanism for this effect is related to the relative sensitivity of free-host DNA to DNAse treatment and to the relative insensitivity of virus capsid-protected DNA to DNAse treatment. We have found that human plasma is replete with free DNA and RNA species and thus opted to examine the potential of DNAse and RNAse treatment to isolate viral nucleic acids. We inoculated human plasma with Adenovirus Type 17 and examined the effects of filtration, DNAse treatment and RNAse treatment on Adenovirus Type 17 DNA and on human β-actin genomic DNA and mRNA, using (RT)-PCR. In Figure 1, we demonstrate that filtration, DNAse and RNAse treatment increases the efficiency of amplification of Adenovirus Type 17 DNA, whereas this combined treatment eliminates amplification of genomic β-actin (upper band) and β-actin cDNA (lower band). Although the addition of RNAse alone has little effect on the β-actin mRNA, the combination of DNAse and RNAse completely eliminates β-actin mRNA. We postulate that this finding may be related to the protection of RNA species by DNA:RNA hybrids. Nevertheless, these data demonstrate that we are able to remove host β-actin DNA and mRNA without degrading Adenovirus Type 17 DNA.

### 5'-VVVVVVVVAA-3' Primers Enable Random Multiplex Amplification of Plasmid DNA

We next designed a mixture of random primers that would enable the priming and amplification of all DNA or cDNA molecules, but that would be resistant to primer-dimer amplification. We chose 10 base pairs as a compromise length to simultaneously optimize hybridization and possible binding sites. *Taq *polymerase requires that the three 3' bases of a primer be 100% complementary in order to enable efficient polymerization. We postulated that "locking" the 3' end of the primers with 2 adenosines and not incorporating any thymidines into the upstream 5' portion of the primers would prevent primer-dimer amplification (*i.e., *5'-VVVVVVVVAA-3'; where V = A, G or C). This primer design will limit the frequency of binding sites within a particular viral genome, but using the EditSeq DNA Editing application (DNAStar), we observed that primers within this mixture will bind every 200–500 bp in both orientations in the 19 viral genomes that were examined, thus providing ample hybridization and amplification potential.

**Figure 2:**
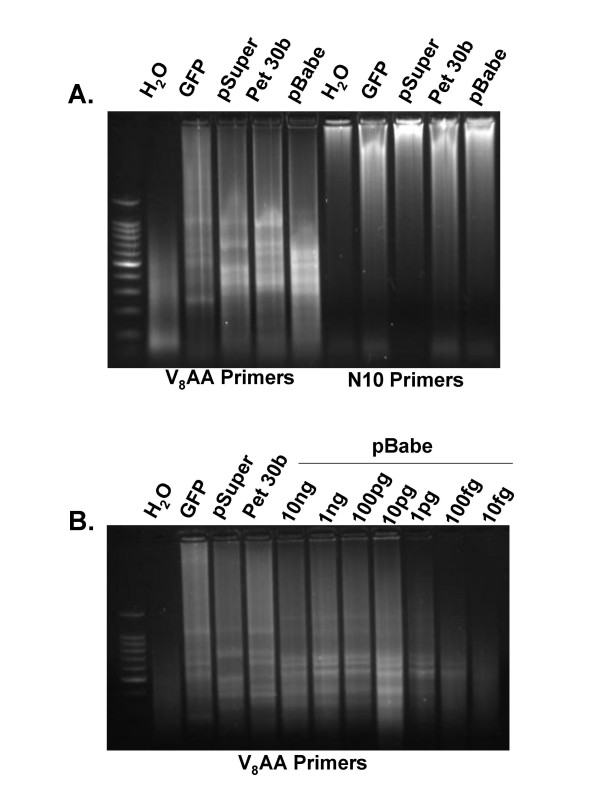
**Random Multiplex PCR With 5'-VVVVVVVVAA-3' Primers Amplifies Circular Plasmid DNA**. **A**. The indicated plasmids were amplified using random multiplex PCR with either 5'-VVVVVVVVAA-3' or 5'-NNNNNNNNNN-3' primers as indicated in the Methods section. **B**. The pBabe plasmid was diluted to the indicated amounts and amplified using random multiplex PCR with 5'-VVVVVVVVAA-3' primers.

As a first test of these random primers we employed DNA plasmids of known sequence. We found that this mixture of primers enabled a continuum of discrete PCR products to be amplified from 4 different circular DNA plasmids (Figure 2A). We shot-gun cloned these PCR products and confirmed that the amplified DNA had derived from the source plasmids (*data not shown*). In contrast, primers consisting of the sequence 5'-NNNNNNNNNN-3' (where N = A, T, G, or C) did not enable the production of discrete or clonable PCR amplicons (Figure 2A). We then examined the ability of the V8AA primers to PCR amplify the plasmid, pBabe, and found that we could amplify as little as 100 fg (Figure 2B).

### 5'-VVVVVVVVAA-3' Primers Allow For the Random Multiplex Amplification of DNA from Human Plasma Inoculated with Adenovirus Type 17

**Figure 3:**
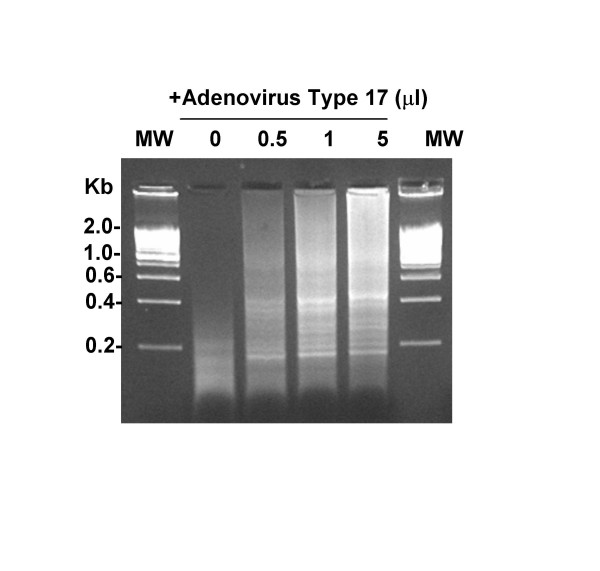
**5'-VVVVVVVVAA-3' Primers Enable Random Multiplex Amplification of DNA from Human Plasma Inoculated with Adenovirus Type 17**. 1 ml of human plasma was inoculated with 0–5 μl of suspended Adenovirus Type 17 (ATCC), filtered and incubated for 2 hours with nucleases. Remaining nucleic acids were purified with the QiaAmp UltrasSens Virus Kit (Qiagen) and subjected to the random multiplex PCR with 5'-VVVVVVVVAA-5' primers as detailed in the Methods section. Amplicons were then visualized on an ethidium bromide impregnated agarose gel.

**Figure 4:**
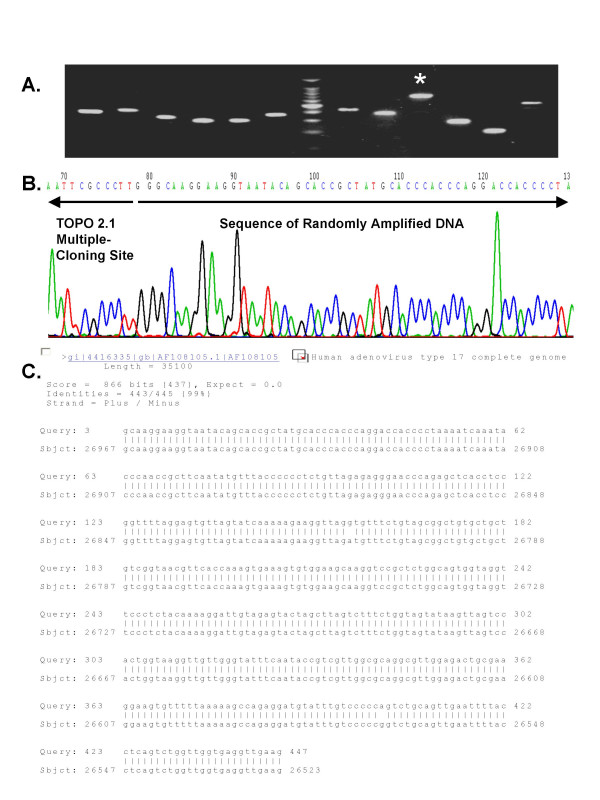
**PCR Screening and Sequencing of Randomly Amplified Adenovirus Type 17 DNA**. Randomly amplified DNA from Adenovirus 17-infected plasma was shot-gun ligated into pCR 2.1-TOPO and competent E. coli were transformed. Resultant colonies were screened for the presence of recombinant plasmid DNA (**A**) and plasmid DNA from positive colonies was then purified and sequenced (**B**). Sequence data from recombinant plasmid #9 (see *) was aligned to all sequence data in the Non-Redundant NCBI Database using the NCBI Nucleotide-Nucleotide BLAST (blastn) Search Algorithm (version 2.2.8) (**C**).

We inoculated 1 ml of human plasma with Adenovirus Type 17. The plasma was then filtered (220 nm filter) and incubated for 2 hours with nucleases. Remaining nucleic acids were purified and PCR amplified using the 5'-VVVVVVVVAA-3' primers. We found that this mixture of primers enabled discrete PCR products to be amplified in the human plasma that had been inoculated with Adenovirus Type 17 and treated with filtration, DNAse and RNase treatment (Figure 3). Importantly, we observed only minimal amplification products in the un-inoculated plasma. We next shot-gun cloned the PCR amplicons into the TOPO 2.1 cloning vector and PCR screened colonies from *E. coli *that had been transformed with mixtures of recombined plasmids. In the un-inoculated plasma samples, we were unable to clone and sequence the amplicons (*data not shown*). In subsequent experiments we have had variable success obtaining random multiplex PCR amplification from human plasma and have obtained only 7 sequences in total from un-inoculated plasma. These sequences did not align with any known sequences in NCBI Non-Redundant database (Blastn searches were conducted.) However, when we shot-gun cloned the DNA amplified from the Adenovirus 17-inoculated human plasma, we obtained 12 recombined vector clones, and all clones matched to Adenovirus Type 17 (see representative example, Figure 4). These data demonstrate that the combination of filtration, DNAse/RNAse treatment and V8AAmer Random Multiplex PCR allows for the detection and identification of Adenovirus Type 17 without using any virus-specific reagents such as primers, antibodies or microarray chips.

### Sensitivity of the Random Multiplex PCR Using 5'-VVVVVVVVAA-3' Primers

The suspension of Adenovirus Type 17 received from the ATCC had not been quantified and the sensitivity of the random multiplex PCR method thus was uncertain. In order to generate a standard curve, we conducted real-time PCR of a known concentration of PCR-amplified Adenovirus Type 17 genomic template using Adenovirus Type 17-specific primers and found that we could detect as little as 10 copies/ml of template (*data not shown*). We then subjected the ATCC Adenovirus Type 17 suspension from the ATCC to real-time PCR using the same Adenovirus Type 17-specific primers and found that we could readily detect the genomic DNA (*data not shown*). Based on the real-time PCR generated from the known concentrations of Adenovirus Type 17 genomic template, we generated a standard curve plotting the concentration of Adenovirus Type 17 genome equivalents *versus *the cycle number when fluorescence passed the pre-set threshold (Ct; Figure 4A). Based on this standard curve, we determined that the stock solution of Adenovirus Type 17 contained 1 × 10^7 ^genome equivalents/ml. We then titrated down the concentration of virus in human plasma and conducted the combination of filtration, DNAse/RNAse treatment and random multiplex PCR using the 5'-VVVVVVVVAA-3' primers. We found that the random multiplex (RT)-PCR method is sensitive to 1000 genome equivalents/ml (Figure 4B).

### Application of Random Multiplex PCR to RNA Viruses

**Figure 5:**
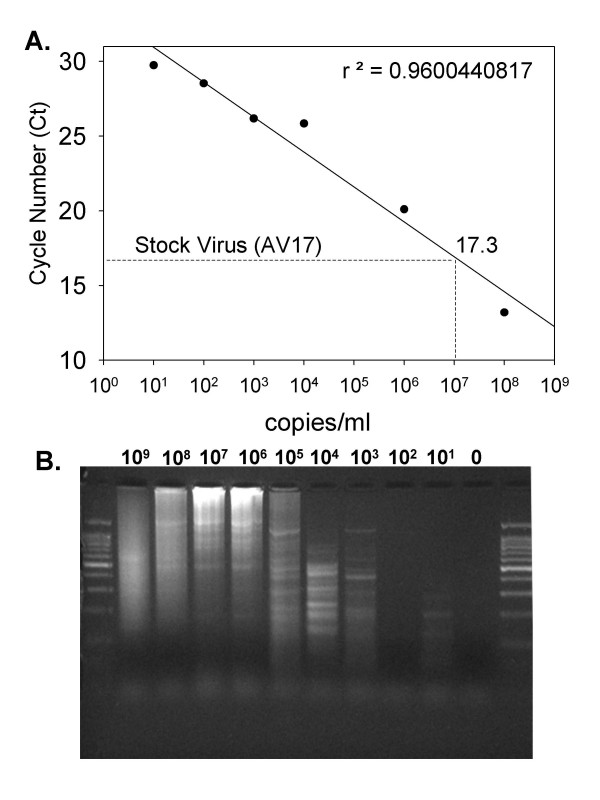
**Real-Time PCR Quantitation of Adenovirus Type 17 DNA Template**. **A**. PCR-amplified genomic DNA from Adenovirus Type 17 was quantitated by spectrophotometry (OD 260 nm) and the genome equivalents/ml calculated based on Avogadro's number (6.02205 × 10^23^/mol). Real-time PCR was conducted with Adenovirus Type 17 specific primers using 10^9 ^to 10^1 ^genome equivalents/ml as template and then using 1 μl of the stock solution of Adenovirus Type 17 as template. A standard curve was produced based on the threshold cycle number of the tested concentrations of Adenovirus Type 17 DNA template and plotted compared to the threshold cycle number (C_T_) of the stock solution of Adenovirus Type 17. **B**. The indicated genome equivalents of Adenovirus Type 17 were added to 1 ml of human plasma, treated with 220 nm filtration and DNAse/RNAse digestion. Total nucleic acids then were purified, amplified by random multiplex PCR (50 cycles) and sequenced as described in the Methods section.

**Figure 6:**
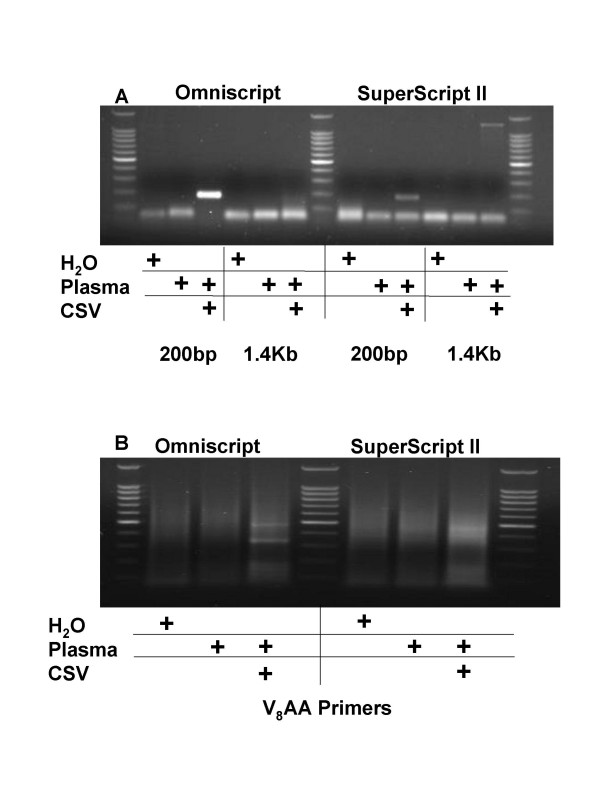
**5'-VVVVVVVVAA-3' Primers Enable Random Multiplex Amplification of Viral RNA from Human Plasma Inoculated with Coxsackie Virus A7**. **A**. 1 ml of human plasma was inoculated with 5 μl of suspended Coxsackie Virus A7 (ATCC), filtered and incubated for 2 hours with nucleases. Remaining nucleic acids were purified with the QiaAmp UltrasSens Virus Kit (Qiagen) and subjected to first strand cDNA synthesis using either Omniscript or Superscript reverse transcriptase. Virus-specific primers were used to amplify either a 200 bp or a 1.4 Kb portion of the viral genome. **B**. The same samples were then subjected to random multiplex PCR with 5'-VVVVVVVVAA-5' primers as detailed in the Methods section. Amplicons were then visualized on an ethidium bromide impregnated agarose gel.

**Figure 7:**
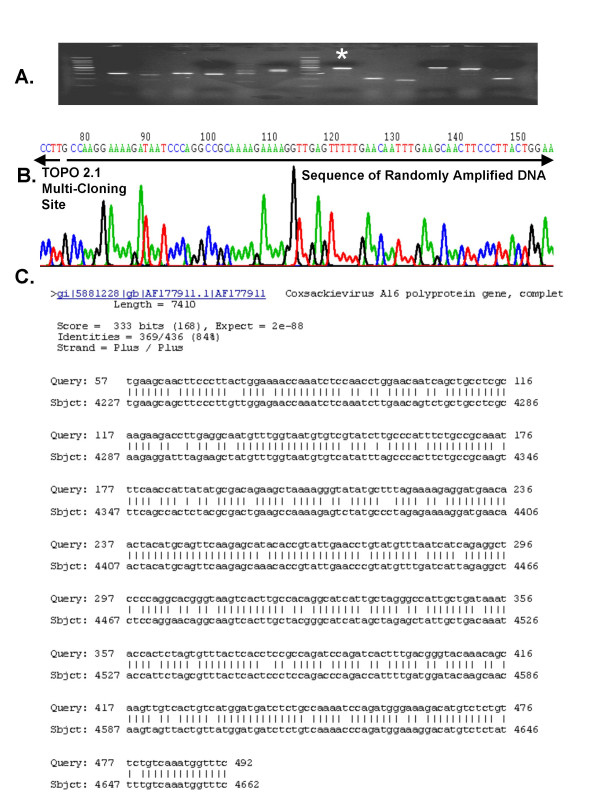
**PCR Screening and Sequencing of Randomly Amplified Coxsackie Virus A7 cDNA**. Randomly amplified cDNA from Coxsackie Virus A7-infected plasma was shot-gun ligated into pCR 2.1-TOPO and competent E. coli were transformed. Resultant colonies were screened for the presence of recombinant plasmid DNA **(A) **and plasmid DNA from positive colonies was then purified and sequenced **(B)**. Sequence data from recombinant plasmid #7 (see *) was aligned to all sequence data in the Non-Redundant NCBI Database using the NCBI Nucleotide-Nucleotide BLAST (blastn) Search Algorithm (version 2.2.8).

We postulated that the random multiplex PCR method would also detect RNA viruses if the viral nucleic acids were first subjected to 1^st ^strand cDNA synthesis. We inoculated human plasma with the RNA virus Coxsackievirus A7 (CSV A7) and then filtered, treated with DNAse and RNAse, purified the remaining nucleic acids and conducted (RT)-PCR, using two commercially available reverse transcriptases (Omniscript and SuperScript II) and CSV A7 specific primers separated by either 200 bp or 1.4 Kb. We found that whereas Omniscript RT was more efficient at amplifying 200 bp cDNA species, SuperScript II RT was more efficient at amplifying 1.4 Kb fragments (Figure 5A). Next, we used the 5'-VVVVVVVVAA-3' primers to random multiplex PCR amplify the cDNA and observed distinct albeit faint amplicons (Figure 5B). We cloned the amplification products and found that the amplified DNA products matched the sequence of the cloned RNA genomes as determined by BlastN alignment searches of the NCBI Non-Redundant database (Figure 6). We were surprised that the cloned DNA from Coxsackievirus A7 aligned with the known sequence for Coxsackievirus A16 until we learned that the Coxsackievirus A7 strain had not been fully sequenced and is thus not in the NCBI Non-Redundant Database (search terms Coxsackievirus AND A7). Accordingly, we had amplified portions of the Coxsackievirus A7 genome that had not previously been sequenced (Figure 7). We also inoculated human plasma with Respiratory Syncytial Virus B and were able to amplify and identify genomic RNA using the 5'-VVVVVVVVAA-3' primers (*data not shown*). However, the PCR amplification efficiency was poor in both RNA viruses relative to the DNA virus, AV17, and we suspect that this may be related to the efficiency of the reverse transcriptases or the stability of the RNA.

## Conclusion

In November 2002, an outbreak of atypical pneumonia occurred in Guangdong Province, People's Republic of China [13,14]. The disease did not respond to empirical antibiotic treatment, and no known bacterial or viral pathogens were identified using serological and (RT)-PCR analyses. The disorder was called Severe Acute Respiratory Syndrome (SARS), and by July 2003 it had caused 8439 reported cases and 812 fatalities [13,14]. In an effort to identify the causative agent of SARS, the WHO SARS Aetiology Study Group coordinated the distribution of information and infected materials and the analyses of the data amongst eleven separate laboratories [14]. Researchers cultured fetal rhesus kidney cells with infected nasopharyngeal aspirates and then conducted random RT-PCR using the primer, 5'-GCCGGAGCTCTGCAGAATTCNNNNNN-3' for reverse transcription, and 5'-GCCGGAGCTCTGCAGAATTC-3' for PCR. Unique PCR products in the infected cell preparation were cloned and sequenced, and 57% homology was found with bovine Coronavirus[13]. RT-PCR of hundreds of infected clinical specimens, using primers specific for the novel virus, confirmed the identification [13,14]. While this approach was successful, it is worthwhile to note that it required 5 months and 11 laboratories to identify the virus. By contrast, the procedures described herein are – at least theoretically – capable of identifying an unknown virus without the use of specially designed primers. Furthermore, is possible to at least partially identify an unknown agent within 2–3 days in one laboratory without intermediate culture steps.

In the current study, we have detected and identified 3 separate viruses (*i.e., *Adenovirus Type 17, Coxsackievirus A7, Respiratory Syncytial Virus B), using the novel approach of random multiplex (RT)-PCR of purified viral nucleic acids. The detection was accomplished in 7 hours using only a micro-centrifuge and PCR machine and did not require any virus-specific reagents. The sensitivity is ~1000 genome equivalents/ml for Adenovirus Type 17 which is not as sensitive as virus-specific PCR (<50 genome equivalents/ml) but should be adequate for the detection of free virus in untreated individuals. Coupled with the potential for direct sequence information, we are confident that this approach may prove useful for our defense against acts of bioterrorism, as well as for the detection and characterization of novel viruses in blood products and in patients displaying hallmarks of infection (*e.g., *leukocytosis, fever).

Although we believe that the method is adequate in current form to test clinical specimens in a randomized, blinded diagnostic trial, we consider that certain alternative approaches may yield a method that has improved sensitivity and specificity. In particular, we intend to decrease the background amplification that has been variably observed in order to prevent false positives and to increase sensitivity by applying SYBR green fluorescence for DNA detection. Finally, we will apply the optimized approach to blood specimens from virally infected patients in order to detect unsuspected variables and to validate the method within the clinical environment.

Our long-term goal is to provide emergency room and primary care physicians with the ability to universally detect viruses in human plasma. The rationale is, in part, that if an emerging or recombined pathogenic virus were intentionally released into a population, then the ability of physicians to detect the pathogen will be crucial for containment. A second and related application of this technique involves identification of previously undescribed – but naturally occurring – viruses in patients suffering with idiopathic disease states of possible viral etiology. A third application of these procedures may be in the screening of blood bank blood just for the presence of virus. Although the precise identification of the virus requires cloning and sequencing, the amplification pattern will provide an immediate signature indicating the presence of virus. Most clinical laboratories that service emergency rooms and primary care clinics possess the ability to conduct RT-PCR for known viruses such as HIV, and the unmodified random multiplex (RT)-PCR method does not require any further technical expertise. In conclusion, the optimization and testing of the random multiplex (RT)-PCR technology should allow for the development of a universal virus detection assay that will vastly improve our collective defense against viral bioweapons and against emerging, heretofore unidentified natural viruses.

## Methods

### PCR of plasmids using 5'-VVVVVVVVAA-3' and 5'-NNNNNNNNNN-3' primers

Plasmids (10 ng each) pEGFP (Clontech), pSuperNeo (Oligoengine), Pet 30b (Calbiochem), and pBabe (Addgene) were added to a PCR mix containing (per reaction): 1 μl of 40 μM of one of the primers indicated (Oligos Etc), 2.5 μl 10× PCR buffer (Promega), 5 μl (5 mM) MgCl_2_, 1 μl (5 mM) dNTPs, 14 μl water, and 0.6 μl Taq polymerase (Promega). PCR was performed according to the following protocol on an Eppendorf Mastercycler Gradient: 95°C-2 minutes and then 94°C-30 seconds, 33°C-59 seconds, 34°C-6 seconds (increase in increments of 1°C for 6 seconds each up to 57°C for 6 seconds), 58°C-2 minutes (+ .3°/s), repeat 50 times, 37°C-5 minutes, 65°C-5 minutes (+ .3°/s), and then hold at 4°C. Products were run on a 1% agarose gel containing ethidium bromide and images were captured on a UVP, Bio-Doc It, gel documentation system.

### Viral samples

Adenovirus Type 17 was purchased from ATCC (VR-1094). This virus is a dsDNA virus. The source was conjunctival scraping from a 1-year old female, Saudi Arabia, 1955. Coxsackievirus A7 W.P. Parker was purchased from ATCC (VR-166). This virus is a ssRNA virus. The source was stool of a 7-year old with fever, headache, sore throat, stiff neck, and back pain, New York, 1949. Respiratory syncytial virus was purchased from ATCC (VR-1580). This virus is a ssRNA, linear virus. The source was the respiratory secretions from a child with acute respiratory disease.

### Blood Plasma Isolation and Viral Separation

10 ml of heparinized whole blood was placed into a 15 ml Falcon tube (BD Biosciences) and centrifuged at 1700 × g for 10 minutes. 250 μl of plasma layer was added to 250 μl room temperature 1× PBS and placed in a SPIN-X .22 micron, 2 ml centrifuge tube (Costar). Plasma was then inoculated with virus and the mixture was centrifuged at 2500 × g for 15 minutes. If any of the mixture remained above the filter, centrifugation was continued at higher speeds until all plasma had passed the filter into the collection tube. 500 μl of PBS was added to the filtered mixture and treated with 7 μl each of un-diluted Riboshredder (Epicentre) and Omnicleave (Epicentre). Treatment of samples with the RNAse and DNAse should cleave any DNA or RNA not protected by viral capsid. All samples were placed in a Thermomixer R (Eppendorf) at 37°C and shaken for 15 seconds every 3 minutes for 2 hours. Plasma was then ready for viral isolation.

### Isolation of Virus from Plasma

1 ml of blood plasma ± virus was cooled to room temperature and placed into a 2 ml tube. Viral isolation was carried out according to the manufacturer's protocol (Qiagen). Viral DNA or RNA was eluted off the column in a final volume of 30 μl elution buffer. This product was either used directly for random PCR or used in the reverse transcription reaction.

### Reverse Transcription of Viral RNA

Reverse transcription was carried out according to the manufacturer's protocol either with the Omniscript RT (Qiagen) or SuperScript II RT (Invitrogen). The maximum amount of viral RNA was used in place of water for both reactions.

### Isolation and cloning of random PCR products

The PCR of viral DNA isolated from whole blood was done according to the method described above utilizing the 5'-VVVVVVVVAA-3' primers. Products were run on a 1% agarose gel containing ethidium bromide. The bands which differed from the water control were excised and the DNA was extracted using the Qiaquick gel extraction kit (Qiagen). DNA was eluted off in a final volume of 20 μl elution buffer. The DNA was cloned into the TOPO cloning vector (Invitrogen) following the manufacturer's protocol and transformation was carried out in TOP 10 chemically competent *E. coli*. Resulting colonies were picked randomly and mixed with 20 μl PBS. 1 μl of this mixture was used in the PCR reaction using the universal M13 forward and reverse (1 μl of 10 μM) primers and 2 × PCR master mix (Promega). The following parameters were used for colony screening: 95°C-2 minutes, and then 36 cycles of 94°C-30 seconds, 55°C-30 seconds, 72°C-1 minute. PCR products were run out on a 1% agarose gel containing ethidium bromide. Those colonies which yielded products greater than 200 bp were grown in LB broth overnight and the DNA was isolated according to the manufacturer's protocol (Qiagen) eluting the DNA off in a final volume of 25 μl. DNA was sequenced using the universal M13 forward and reverse primers at the University of Louisville's core sequencing facility. Resulting sequences were screened using the BLAST program on the NCBI web site to determine sequence origin.

### PCR from Viral DNA or cDNA with Virus-specific Primers

PCR was carried out with either viral DNA or cDNA using a 2× PCR Master Mix (Promega) and 1 μl of viral DNA. 1 μl of 10 μM primers was used for each reaction. The total volume was equal to 25 μl per reaction. The following parameters were used for viral-specific amplification: 94°C-5 minutes, 94°C-30 seconds, 55°C-30 seconds, 72°C-2 minutes, repeat 45 times, 72°C-7 minutes.

Virus-Specific Primers (standard PCR)

For generation of 200 bp fragment:

Coxsackievirus forward primer (923): 5'-TTATCAGAGATGGCAGCACC-3'

Coxsackievirus reverse primer (1105C): 5'-CTTGTCCACCGCTGTAGCCT-3'

For generation of 1.4 Kb fragment:

Coxsackievirus forward primer (923): 5'-TTATCAGAGATGGCAGCACC-3'

Coxsackievirus reverse primer (2161C): 5'-CATTGCCTGCATTCTGTTGG-3'

Virus-Specific Primers (For Real Time PCR)

Coxsackievirus forward primer (937): 5'-AGCACCACTGCAATCACCGA-3'

Coxsackievirus reverse primer (1094C): 5'-CTGTAGCGTCAGTGTCAGGA-3'

Adenovirus forward primer (5770): 5'-TGTAGGTGTAGGCCACGTGA-3'

Adenovirus reverse primer (6084C): 5'-TCGCCAAGCTTCTCTCCAAC-3'

Template Production Primers:

Adenovirus forward primer (5492): 5'-CTCTTACCTCGCGTCTCCAT-3'

Adenovirus reverse primer (6084C): 5'-TCGCCAAGCTTCTCTCCAAC-3'

Coxsackievirus forward primer (923): 5'-TTATCAGAGATGGCAGCACC-3'

Coxsackievirus reverse primer (1105C): 5'-CTTGTCCACCGCTGTAGCCT-3

### Generating viral DNA templates for real time PCR

Viral DNA isolated from whole blood plasma was used to generate a template for use in real time PCR. 2 μl of DNA from the virus was used with Platinum PCR Master Mix (Invitrogen) and 2 μl of a 10 μM solution of each of the template primers outlined above. The total volume of the reaction was 50 μl. The same PCR parameters were used as were used for viral-specific amplification. The resulting products were run on a 1% agarose gel containing ethidium bromide. The bands were excised and DNA was eluted according to manufacturer's instructions (Qiagen).

### Real Time PCR

All real time PCR samples were run on a Cepheid Smart Cycler. OmniMix Hs Reagent (Cephied, packged by Takara) was re-suspended in 50 μl of water, which was sufficient for two PCR reactions. 1 μl of the stock virus and 1 μl of each of the template primers (10 μM) were added and mixed. Samples were run at 95°C for 30 sec, 54°C for 15 sec, and 72°C for 20 sec for a total of 40 cycles.

### Determination of viral molecules in viral template

The molecular weight was determined for each of the dsDNA templates generated by PCR. The OD of the resultant DNA was also recorded. The OD, in grams/μl was divided by the molecular weight of the product and the result was equal to moles/μl. This number was then multiplied by Avogadro's number (6.022 × 10^23 ^molecules/mole) and the resulting number is equal to the number of viral DNA molecules per microliter.

## Competing interests

The author(s) declare that they have no competing interests.

## Authors' contributions

ALC: Conducted multiplex (RT)-PCR, cloning, virus-specific PCR, template production and sequencing.

JS: Optimized the random multiplex (RT)-PCR of RNA viruses.

ST: Phlebotomy, serum preparation and assisted with the initial development of the 5-VVVVVVVVAA-3' primers.

JWE: Provided scientific input regarding the universal application of this approach and assisted with the optimization of the random multiplex (RT)-PCR of RNA viruses.

JC: Conceived and directed the entire project. Conducted initial multiplex PCR for AV17.

All authors read and approved the final manuscript.
